# Development of a novel observer-reported outcome measure for the assessment of Respiratory Syncytial Virus (RSV) infection symptoms in pediatric clinical trials

**DOI:** 10.1186/s41687-018-0034-9

**Published:** 2018-02-21

**Authors:** Sandy Lewis, Carla DeMuro, Stan L. Block, Shelly Senders, Paul Wisman, Seth Toback, Jason W. Chien, Valerie Williams

**Affiliations:** 1 RTI Health Solutions, 200 Park Offices Drive, Research Triangle Park, NC 27709 USA; 2 Kentucky Pediatric & Adult Research, Bardstown, KY USA; 3 Senders Pediatrics, South Euclid, OH USA; 4 Pediatric Research of Charlottesville, Charlottesville, VA USA; 5 biomerieux, Durham, NC USA; 6 Gilead Sciences Inc., Foster City, CA USA

**Keywords:** Respiratory syncytial virus, Clinical outcomes assessment, Observer-reported outcome, Caregiver diary

## Abstract

**Background:**

Respiratory syncytial virus (RSV) is a seasonal infection affecting most children by 2 years of age and the leading cause of lower respiratory tract infection requiring hospitalization in infants. Novel antiviral medications are in development to improve the clinical outcomes of RSV; however, no clinical outcome assessments (COAs) for RSV have been developed in alignment with the United States Food and Drug Administration patient-reported outcome guidance to assist in the evaluation of new therapies. To address this need, an observer-reported outcome (ObsRO) measure designed to assess observable RSV symptoms was created.

**Methods:**

The literature was reviewed to evaluate existing COAs and identify constructs of interest. Individual caregiver interviews elicited concepts that informed item development, and candidate items were subsequently evaluated in two rounds of cognitive testing. Separate cohorts of caregivers of RSV-infected nonhospitalized and hospitalized infants participated. Therapeutic-area experts provided input throughout the instrument development process.

**Results:**

Caregivers of 39 children < 24 months old with RSV (31 nonhospitalized, 8 hospitalized) participated in in-depth, individual interviews during concept elicitation and cognitive debriefing, resulting in 21 concepts identified as potentially observable and relevant to young children with RSV. The item pool was reduced to 12 cardinal symptoms and behavior impacts reported to be directly observable by caregivers, with 10 daytime and 9 nighttime symptoms to capture diurnal variation in severity.

**Conclusions:**

The RSV Caregiver Diary assesses RSV symptom severity and change from the parent or caregiver perspective in a standardized manner to measure treatment benefit. Following psychometric evaluation and refinement, this tool is expected to be suitable for assisting in the clinical development of RSV therapeutics.

**Electronic supplementary material:**

The online version of this article (10.1186/s41687-018-0034-9) contains supplementary material, which is available to authorized users.

## Background

Clinical outcome assessments (COAs) are generally designed to assess how a patient feels or functions [1]. This type of information is typically most accurately captured based upon self-report. However, in populations where self-report is not possible or unreliable, (e.g., very young children, patients with significant cognitive deficit), the United States (US) Food and Drug Administration (FDA) encourages observer reporting of those signs, symptoms, events, or behaviors that can be clearly observed and reported [2]. In lieu of self-report, observer-reported outcomes measures (ObsRO) provide invaluable insight into the patient experience of a disease or therapy, that may be otherwise unobtainable, and complement existing clinical indices. COA data are typically collected via standardized questionnaires designed to measure an explicit concept or construct such as symptoms, activity limitations, and health status/health-related quality of life, and are commonly utilized in randomized controlled trials [3]. Regulatory guidance recommends rigorous development and validation for any COAs used in support of medical labeling. Such rigor includes direct input from the population of interest during instrument development.

Respiratory syncytial virus (RSV) is a highly symptomatic, common seasonal infection that affects most children by the age of 2 years and is the leading cause of lower respiratory tract infection requiring hospitalization among infants and young children [4]. Novel antiviral medications and vaccines are currently in development to improve the clinical outcome of RSV infections; however, no COAs for RSV disease in infants that meet the requirements of the FDA patient-reported outcome guidance exist. Specifically, existing measures lack evidence of content validity, based on input from patients or caregivers of infants/young children with acute RSV. Further, psychometric rigor is limited with respect to the outpatient measurement of RSV symptom severity in infants and young children with acute RSV infection. Historically, clinical studies of RSV-infected infants have relied on symptom scores or other measures, none of which have been validated to be appropriate as clinical trial endpoints. To address this need, an ObsRO measure based on caregiver report of observable RSV-associated symptoms in young children was developed in alignment with the US FDA guidance for use in a clinical trial setting.

## Methods

The Gilead RSV Caregiver Diary (GRCD) was developed based upon a targeted review of the literature and existing measures, in-depth interviews with caregivers, and expert input.

Caregivers were defined as parents or primary caregivers of children with laboratory-diagnosed RSV infection 24 months of age or younger from five primary care sites within four US states (KY, VA, GA, OH). Before any participant contact, this project was reviewed and approved by an institutional review board and all procedures were conducted in accordance with the ethical standards of the 1964 Helsinki declaration and its later amendments. Written informed consent was obtained from all participants prior to interview recruitment. Entry criteria are detailed within the section describing the concept elicitation methods. These same criteria were utilized during the cognitive debriefing stage.

### Literature Review

A targeted literature and instrument review of available ObsRO and clinician-reported outcome (ClinRO) measures was conducted to better understand concepts relevant to the assessment of symptoms of RSV infection. Relevant literature was identified utilizing PubMed. Global terms related to RSV included “Respiratory syncytial virus;” “Parent OR Caregiver OR Carer;” “Scale OR Measure OR Checklist OR Instrument OR Questionnaire OR Survey OR Caregiver rated OR Observer rated.” A set of inclusion criteria for the PubMed portion of the literature search included the following:

▪ Clinical trials, observational studies, longitudinal studies, naturalistic studies, cross-sectional studies, retrospective or prospective cohort analyses, systematic literature reviews, surveys, or instrument validation studies

▪ Studies published in English

▪ Articles published since 2003; including use of full reference list of all articles selected for review

### In-depth Interviews

#### Concept Elicitation

Concept elicitation interviews were conducted with adult caregivers of children with a laboratory-diagnosed, symptomatic RSV infection. These in-depth, individual interviews were conducted to identify relevant concepts from the caregiver perspective to inform the development of draft items for a new ObsRO instrument. Participants meeting predefined study criteria were recruited by outpatient pediatric clinical research sites during a single RSV season in December 2013. During the course of routine clinical care, trained site staff identified infants and young children < 24 months of age who tested positive for RSV (with a rapid antigen detection kit) through standard-of-care treatment in the office before confirming the remaining study eligibility criteria. To be eligible for participation, an individual must have been the caregiver of a child 24 months or younger at the time of the screening visit who was at least 28 weeks gestational age at birth seeking their first health care visit for a physician-diagnosed acute respiratory tract infection of ≤5 days duration. Full study inclusion and exclusion criteria are listed in the Additional file 1.

Interviews were conducted following a semi-structured discussion guide that was developed based on the information from the targeted instrument review, as well as from clinical physician (SB, PW) input. All participating physicians are practicing pediatricians who routinely treat children with RSV infection. Saturation is defined in the FDA PRO Guidance as “the point when no new relevant or important information emerges and collecting additional data will not likely add to the understanding of how patients perceive the concept of interest and the items in the questionnaire.” An assessment was made between and across participants to determine whether additional interviews were needed.

The interviews were audio-recorded and transcribed; transcriptions were then verified through an iterative process of technical and editorial review.

#### Item Generation

Data from the concept elicitation interviews were analyzed utilizing a constant comparative analysis paradigm [5]. These results were then compiled and grouped by emergent themes. Draft questionnaire items were created from observable, highly endorsed thematic concepts that are generalizable across the target patient population and have potential to change with treatment and over time. A fifth-grade reading level was maintained for item development. Response scales for testing included both categorical and numerical scales to determine the most accurate and sensitive options that naturally related to the items.

#### Cognitive Debriefing

To pretest and refine the draft GRCD, two iterative sets of cognitive debriefing interviews were conducted with caregivers of infants and young children with an RSV infection who were not hospitalized during the course of infection. Participants were required to meet the same inclusion criteria as for the concept elicitation interviews. A third set of interviews were then conducted with 8 similar caregivers of infants and young children < 24 months of age who were hospitalized during the course of their RSV infection. For hospitalized patients, interviews included a brief concept elicitation discussion to confirm comprehensiveness of the instrument prior to cognitive debriefing.

Cognitive debriefing interviews were conducted during a single RSV season in 2014. The majority of interviews were conducted via telephone to allow for expedited recruitment, minimize the time before interview, and to accommodate scheduling requests from caregiver participants. Transcripts from both interview modes were compared in terms of quantity and quality of the data collected.

The objectives of the cognitive debriefing interviews were to understand the question-and-answer process used by the caregiver, refine question wording, optimize the response scales, confirm appropriateness of the recall period, and confirm the content validity of the instrument. Development of the discussion guides followed a similar process to that previously described in the concept elicitation section.

Following analysis of the concept elicitation interview data, three pediatricians supporting the measurement development project convened to discuss results of the concept elicitation component of the study and provide insight into the draft item development process. These physicians also reviewed and provided input on the finalized version of the tool.

## Results

### Literature Review

Initial review of the literature focused on identification of caregiver-reported measures of acute RSV symptoms. Only a single measure, the Bronchiolitis Caregiver Diary (BCD) [6], a measure of post-acute RSV bronchiolitis symptoms, was pertinent. Broader review identified two additional instruments, the Canadian Acute Respiratory Illness and Flu Scale (CARIFS) [7] and the Wisconsin Upper Respiratory Symptom Survey (WURSS) [8–10], however, these tools are not specific to RSV or to the infant population. Because no RSV-specific instrument was identified, it was necessary to expand the search to examine ClinRO measures for the purpose of further understanding the symptoms of RSV infection, especially those symptoms of interest to clinicians. Several ClinRO measures were evaluated for the purpose of describing the symptoms and signs of RSV of interest to clinicians:

▪ Gern Disease Severity score [11]

▪ Respiratory Distress Assessment Instrument [12]

▪ Wang Clinical Severity score [13]

▪ Clinical Scoring System [14]

▪ Respiratory Symptom Log [15]

▪ Lower respiratory infection/illness score [16]

Table 1 summarizes the RSV symptoms assessed by the three ObsRO and six ClinRO measures reviewed. The most frequently assessed RSV symptoms were wheezing (in six measures), cough (in five measures), chest recessions/retractions (in four measures), and rhinorrhea/nasal discharge/runny nose (in four measures). Symptoms or symptom consequences measured less frequently included sleep problems, issues related to eating (e.g., poor appetite), sneezing, stopped or plugged nose/nasal congestion, hoarseness, and apnea. Each of these concepts was addressed only once among the nine measures.

**Table 1:** Respiratory Syncytial Virus Symptoms Assessed Using Observer-Reported and Clinician-Reported Measures

RSV Symptom Measure	Wheezing/Auscultatory breath sounds	Cough	Retractions/Chest recessions	Rhinorrhea/Nasal discharge/Runny nose	Respiratory rate/Rapid breathing/Tachypnea	Trouble breathing/Short of breath	Fever	Skin color/Cyanosis	General Condition/ Appearance	Not sleeping well/Sleeping less than normal	Eating less than normal/Poor appetite	Sneeze	Stopped or plugged nose/Nasal congestion	Duration of illness	Hoarseness	Apnea
Observer-reported																
BCD	●	●				●										
CARIFS		●		●			●			●						
WURSS		●		●							●	●	●			
Clinician-reported																
Gern score	●	●	●	●	●		●	●						●	●	●
RDAI	●		●													
Wang Clinical Severity Score	●		●		●				●							
Clinical Scoring System	●		●		●			●	●							
Respiratory Symptom Log	●	●		●		●										
LRI Score									●							

*BCD* Bronchiolitis Caregiver Diary, *CARIFS* Canadian Acute Respiratory Illness and Flu Scale, *LRI* Lower Respiratory Infection/Illness, *RDAI* Respiratory Distress Assessment Instrument, *RSV* respiratory syncytial virus; *WURSS* Wisconsin Upper Respiratory Symptom Survey

None of the measures identified and reviewed were deemed to be fit for purpose when compared in light of regulatory requirements for COAs utilized in support of medical labeling. Evidence of content validity, based on patient (or caregiver) input is one of the key elements required by FDA guidance. Two of the measures reviewed reported qualitative research involving caregivers during the development process (the BCD and CARIFS) but targeted populations other than acute pediatric RSV. Similarly, psychometric analyses for these COAs supported the reliability, validity, and responsiveness of three of the measures (the BCD, CARIFS, and WURSS) but in populations very different from infants with acute RSV.

Given the lack of evidence of content validity coupled with limited review of instrument measurement properties, development of a new COA was supported.

### Patient Input

#### Concept Elicitation

A total of 16 concept elicitation interviews were conducted to confirm the list of symptoms in Table 1 and to help determine the symptoms most relevant for demonstrating the benefit of RSV treatment as well as to identify those that could most accurately be assessed by observer report. The majority of interviews (*n* = 13) were conducted via telephone to accommodate scheduling requests from caregiver participants; three were conducted in person. Participants were between the ages of 21 and 41 years, predominantly white and female, and just over half of the children they were discussing were male with a mean age of 5.5 months (range 2 to 21 months). Participants in this sample spanned a range of educational categories from a high school education through a graduate degree. The time between the office visit when the RSV diagnosis was made and the interview ranged from 2 to 15 days (median 6.7 days). Table 2 presents the combined demographic characteristics collected at screening for the concept elicitation interview participants.

**Table 2:** Characteristics of Interview Participants

Characteristic	Concept Elicitation *N* = 16^a^	Cognitive Debriefing Outpatient *N* = 15^a^	Cognitive Debriefing Inpatient *N* = 8
Mean participant age (range), years	33.9 (21-41)	29 (18-38)	35 (24-42)
Mean child age (range), months	5.5 (2-21)	11 (3-21)	9.9 (4-20)
Participant gender, n (%)			
Male	2 (12.5)	2 (13.3)	2 (25.0)
Female	14 (87.5)	13 (86.7)	6 (75.0)
Child gender, n (%)			
Male	9 (56.3)	8 (53.3)	5 (62.5)
Female	7 (43.8)	7 (46.7)	3 (37.5)
Participant education, n (%) ^a^			
Less than high school	0 (0.0)	0 (0.0)	0 (0.0)
High school or equivalent	4 (25.0)	4 (26.7)	2 (25.0)
Some college	1 (6.3)	5 (33.3)	0 (0.0)
College degree	8 (50.0)	4 (26.7)	3 (37.5)
Professional or advanced degree	2 (12.5)	1 (6.7)	3 (37.5)
Participant race, n (%)			
Asian	3 (18.8)	0 (0.0)	1 (12.5)
Asian/White	2 (12.5)	0 (0.0)	0 (0.0)
Black	1 (6.3)	2 (13.3)	0 (0.0)
Black/White	0 (0.0)	1 (6.7)	0 (0.0)
White	10 (62.5)	12 (80.0)	7 (87.5)
Days from office visit to interview, median (range)	7 (1-15)	9 (2-20)	N/A

Note: Percentages may not equal 100 due to rounding

*N/A* not applicable

^a^One participant did not provide an educational level

Participants were able to easily recall and discuss the course of their child’s illness, including detailed accounts of symptoms exhibited prior to diagnosis, regardless of the amount of elapsed time between disease onset and the interview. However, despite the ease in recall, measurement of symptom severity on a daily basis was strongly endorsed as the most accurate timeframe for symptom assessment to capture change in symptoms over time. Participants generally described change in symptoms over a 7- to 10-day period (including inpatients), noting an initial increase in symptom severity post-diagnosis, a period of about 3-4 days where symptoms were at their most severe, followed by 4-6 days of gradual symptom improvement.

Participants described fluctuations in the severity of some RSV symptoms between daytime and nighttime hours, leading them to endorse the assessment of symptoms over 12-h time periods. Participants generally reported that use of a numerical rating scale (NRS) or a verbal response scale would be equally understandable and easy to use.

At the beginning of each interview, participants were asked to describe the types of signs or symptoms they observed in their child with RSV infection. Participants described early-onset symptoms and those that were observed throughout the course of their child’s illness. Predominant themes arose across five major categories or concepts: symptoms related to breathing problems, cough, fever, congestion, and an additional category related to symptom impacts. A sixth concept category of “other” was included to encompass predominant themes that did not organically fit within the previously identified categories. Table 3 includes a summary of the overarching categories and symptoms identified by caregivers.

**Table 3:** Concepts Elicited From Interview Participants

Observable Symptom	
Breathing Problems	
	Wheezing
	Difficulty breathing
	Rapid or shallow breathing
	Loud, noisy breathing
	Retractions
	Shortness of breath
Cough	
	Cough
	Nighttime cough
	Vomiting after cough
Fever	
	Fever
Congestion	
	Nasal discharge/runny nose
	Congestion (unspecified)
	Nasal congestion/“stopped nose”
Appetite, Activity, Disposition, and Sleep	
	Eating less than normal
	Less active/sleeping more
	Disposition
	Sleeping less
Other	
	Sneezing
	Eyes—red
	Eyes—watery/watering
	Altered coloring

Concept saturation was achieved and documented.

#### Expert Input

Input from board-certified fellows of the American Academy of Pediatrics, practicing pediatric medicine for at least 25 years in community-based settings, supported both the findings from the literature review and concept elicitation data. Of the 21 observable symptoms reviewed, 12 were selected (Table 4) for item-generation activities based on the combination of caregiver report and physician input. Based on caregiver descriptions, some symptoms seemed to overlap with others and therefore were combined during item development (i.e., difficulty breathing was best described using a combination of loud, noisy breathing and rapid or shallow breathing; shortness of breath was combined with rapid or shallow breathing; congestion (unspecified) was better described as loud, noisy breathing). A small number of observations (eyes red, eyes watery/watering, altered coloring) were not developed into diary items due to infrequent participant reports. Other symptoms were not included due to the potential misunderstanding of the concept (e.g., actual vomiting after cough not distinguished from expulsion of phlegm) or difficulty with interpretation/difficulty with observation (e.g., retractions are typically noted upon clinical observation as opposed to being caregiver identified and reported). Finally, while sneezing was endorsed by nearly half of the participants (7 of 16), the expert physicians noted that this symptom was neither a cardinal symptom nor routinely seen among patients with RSV infection; sneezing was, therefore, not included among the concepts forming the basis for the new diary.

**Table 4:** Respiratory Syncytial Virus Infection: Concept List by Predominant Theme

Concepts	Observable Symptoms
Breathing problems	▪ Wheezing ▪ Rapid or shallow breathing ▪ Loud, noisy breathing
Cough	▪ Daytime cough ▪ Nighttime cough
Fever	▪ Fever
Congestion	▪ Nasal discharge/runny nose ▪ Nasal congestion/stuffy nose
Symptom impacts	▪ Activity ▪ Appetite ▪ Disposition ▪ Sleep

#### Item Generation

Based on the concept elicitation results, following a structured set of item generation principles (for example, succinctly worded items, items expected to demonstrate change over time, etc.), a long-list questionnaire, based on the 12 selected symptoms, was drafted. A pool of 23 daytime and 20 overnight questions representing alternate wording options addressing the 12 selected symptoms was generated with both NRS and verbal rating scale options. Most of the daytime and overnight items contained similar concepts except for a few that were more relevant during the day (e.g., activity). Further, concepts selected were strongly endorsed by caregivers as clearly observable signs associated with RSV infection.

#### Cognitive Debriefing

A total of 15 individual cognitive debriefing interviews were conducted with caregivers of nonhospitalized children diagnosed with RSV in two rounds (8 in round 1 and 7 in round 2). Following this initial work, an additional 8 caregivers of hospitalized children diagnosed with RSV infection were interviewed. The interviews were structured to evaluate the caregiver question/answer process, assess comprehension and refine question wording, optimize the response scales, confirm appropriateness of the recall period, confirm the content validity of the items, and confirm there were no missing concepts. Caregivers with nonhospitalized children were similar to those who participated in the concept elicitation interviews; caregivers were between the ages of 18 and 38 years and predominantly white and female. Just over half of the children they were discussing were male with a mean age of 11 months (range 3 to 21 months). Participants in this sample spanned almost the full range of educational categories. The time between the office visit when the RSV diagnosis was made and the interview ranged from 2 to 20 days (median 9 days), a time window during which parents reported having no trouble recalling symptoms. Caregivers of hospitalized children were between the ages of 24 and 42 years and predominantly white and female. Just over half of the children they were discussing were male with a mean age of 9.9 months (range 4 to 20 months). Table 2 summarizes the information collected at screening for the cognitive debriefing interview participants.

Caregivers of nonhospitalized infants across both rounds readily endorsed the majority of items presented within the GRCD as being relevant and important to the measurement of observable symptoms of RSV infection. However, the concept of wheezing was not easily understood by the majority of participants. Despite endorsement during concept elicitation, it became apparent that participants were unable to identify accurately and report observations of wheezing. This finding is consistent with the results of previous qualitative work conducted in caregivers of infants with bronchiolitis [6]. Upon agreement with the physician experts this item was ultimately removed.

In round 1 of the cognitive debriefing, caregivers of nonhospitalized infants and children < 24 months of age readily endorsed the majority of items presented within the draft GRCD as being relevant and important to the measurement of observable symptoms of RSV infection. Minor modifications were made to the instructional text and additional examples were added to a single item to improve clarity. Participant feedback supported use of the verbal rating scale options as they were deemed to be representative responses that better matched how respondents naturally thought about their observations related to their child’s symptoms. Finally, feedback concerning the alternate wording options resulted in deletion of items asking respondents to “rate” the severity of each symptom concept presented.

A reduced pool of 12 daytime and 10 overnight questions were included for further testing in round 2. Participant feedback received during round 2 further informed item reduction based on selection of the most appropriately worded item alternates. An additional item, daytime sleep, was removed as results could prove difficult to interpret because sleeping either more or less could represent an increase in RSV severity and increased sleep would already be captured within the activity item (i.e., no activity), and decreased sleep could also correlate with mood (e.g., increased fussiness). Because this item did not seem to provide additional evaluative information but rather would introduce noise into the overall measure, it was deleted from the final version of the GRCD. Further wording revisions were made to response options associated with three items (sleep, disposition, and activity) to mirror the direction of the other scales and to maintain consistency across the wording in the response scales included in the final version. This wording tested well across similar response options reviewed during the two rounds of testing, which provide further support for the small modification. Finally, while the item assessing fever tested well and was deemed important, participants noted potential value of applying a range of values based on actual temperature for those using a thermometer to monitor fever.

Participant endorsement of items across the two rounds of interviews confirmed content relevance. Participants noted some potentially missing items from the questionnaire. The presence of an ear infection or nosebleed were suggested as well as frequent, runny stools. The impact of ear infection, commonly seen in conjunction with RSV, would likely be captured under other items such as sleep or disposition. As clinical experts confirmed none of the remaining suggestions pertained to common signs or symptoms of RSV infections, they were not appropriate for inclusion in the diary. No additional concepts were noted as missing from the questionnaire, providing evidence to support content validity.

Similar results were garnered from the interviews conducted with caregivers whose children were hospitalized due to RSV infection. All endorsed the items included in the GRCD as easy to understand and relevant to the assessment of observable symptoms of RSV infection. All participants were easily able to select a response from the options that were provided; noting that the options naturally related to the items. Further, there were no missing concepts raised that were relevant for inclusion. Results of these additional cognitive debriefing interviews further support the content validity of the GRCD and demonstrate the relevance of the concepts in a more severe population (i.e., those hospitalized due to RSV infection). These interviews also confirmed that the changes made to the items after round 2 of the initial cognitive debriefing interviews were acceptable and appropriate. This qualitative research produced a developmental version of the GRCD poised for psychometric evaluation. An abbreviated item tracking matrix is included in the Additional file 2.

## Discussion

Changes in legislation over the past several years have led to an increase in the conduct of pediatric clinical trials, and although the FDA PRO guidance acknowledges the importance of PRO measures in pediatric populations and applies the same standards for development, it does not provide specific recommendations to address the challenges of developing tools for use in this younger population. A working group convened by the International Society for Pharmacoeconomics and Outcomes Research sought to address some of these challenges, such as determination of the recommended age for self-report, utilizing children as experts, and instrument design to facilitate accurate reporting (i.e., form design, electronic vs. paper-based) [17]. Results of this research support data collection in pediatric outcomes, however, in this younger age group, self-assessment is not possible and therefore caregiver reported observations were necessary.

## Conclusions

The GRCD was developed in alignment with the FDA PRO guidance following a rigorous and iterative process, thereby filling the need for a valid, reliable, and responsive measure of RSV symptom severity and change after treatment in infants and very young children.

Qualitative research, including a review of the literature and direct expert and caregiver input, culminated in a 19-item, developmental version of the GRCD with 9 overnight questions and 10 daytime questions specific to symptoms that can be directly observed by the caregiver of a child with RSV infection. The items included in the GRCD align with the observable concepts identified in the literature review. The GRCD is designed to be self-administered by the caregiver to gather direct caregiver input, completed twice daily at two separate times with recall periods of overnight symptoms (“in the morning after your child has woken up for the day”) and daytime symptoms (“since your child awoke this morning until you put your child to bed”) in order to capture diurnal variations in symptoms. GRCD items are scored on 5- to 6-point ordinal rating scales assessing symptom severity, with an additional option of “I don’t know” for select items assessing overnight symptoms.

Importantly, to our knowledge, the GRCD is the only ObsRO for RSV disease developed in accordance with the FDA PRO guidance recommendations for use in clinical trials incorporating feedback from the population of interest and capable of assessing change after treatment in a standardized manner and supporting an understanding of treatment benefit for novel RSV therapies. A preliminary psychometric evaluation of the draft GRCD has been conducted and a revised version of the tool has been developed [18]. The post-validation version has been further refined and items deemed redundant where deleted, providing a briefer and more succinct measure appropriate for both clinical trials and the clinical setting.

There are some limitations to this research. Participants were recruited as part of a convenience sample. While some geographic diversity was attempted during site selection, participant recruitment was limited to the Eastern United States. Additionally, while ethnic diversity was attempted, the majority of the participant sample were Caucasian. While caregiver-reported concepts were consistent throughout this sample, the impact of a more diversified sample is unknown.

## Additional files

Additional file 1: Full Study Inclusion/Exclusion Criteria. (DOCX 19 kb)

Additional file 2: Abbreviated Item Tracking Matrix. (DOCX 18 kb)
