# Supplementary material for: Development of a novel observer-reported outcome measure for the assessment of Respiratory Syncytial Virus (RSV) infection symptoms in pediatric clinical trials
Source: J Patient Rep Outcomes. 2018 Feb 21;2:9. doi: 10.1186/s41687-018-0034-9 (PMC5935018; doi:10.1186/s41687-018-0034-9)
Supplement: Supplementary file 1 — Full Study Inclusion/Exclusion Criteria. (DOCX 19 kb) [file 41687_2018_34_MOESM1_ESM.docx]

#### Inclusion Criteria

- Parent/caregiver of a child aged less than 24 months at screening visit.
- Parent/caregiver of a child of at least 28^[[1]](#endnote-1)^ weeks gestational age at birth.
- Parent/caregiver of a child with presence of signs and symptoms indicative of an RSV lower respiratory tract infection (typically rhinitis, tachypnea, wheezing, cough, crackles, use of accessory muscles, and/or nasal flaring) for ≤ 5 days duration at the time of the office visit.^[[2]](#endnote-2)^
- Caregiver’s child with previous RSV infection admitted to the hospital or short stay unity within the past 6 months.^[[3]](#endnote-3)^
- Parent/caregiver of a child with a confirmed positive RSV test. ^[[4]](#endnote-4)^
- Documentation of a confirmed diagnosis of RSV infection during the hospitalization or within 72 hours prior to the child’s hospitalization. ^[[5]](#endnote-5)^

#### Exclusion Criteria

- Caregiver’s child with previous RSV infection experienced respiratory failure requiring endotracheal intubation and mechanical ventilation or tracheal ventilation during the hospitalization. ^[[6]](#endnote-6)^
- Caregiver’s child with previous RSV infection admitted to any pediatric intensive care unit (ICU) for the RSV illness. ^[[7]](#endnote-7)^
- Parent/caregiver of a child who has any chronic renal, hepatic, neurological, hematologic, or metabolic disorder (including diabetes mellitus), [pre-existing lab abnormalities associated with these disorders^[[8]](#endnote-8)^] or any genetic syndrome.
- Parent/caregiver of a child who is currently receiving supplemental oxygen therapy for chronic lung disease or congenital heart disease.
- Parent/caregiver of a child who received a diagnosis of any oncologic disease [treated with chemotherapy in the prior 12 months^[[9]](#endnote-9)^].
- Parent/caregiver of a child who has a known immunodeficiency disorder.
- Parent/caregiver of a child who has participated in a study with any investigational medicinal product [in the previous 28 days^[[10]](#endnote-10)^] [during the course of RSV infection (i.e., when symptoms started, during hospitalization, and/or through recovery^[[11]](#endnote-11)^].
- Parent/caregiver of a child who was hospitalized within the 28 days prior to the screening visit. ^[[12]](#endnote-12)^
- Parent/caregiver of a child who was hospitalized at any time between screening visit and the date of the interview. ^[[13]](#endnote-13)^

1. Revised to 35 weeks for in-patient study [↑](#endnote-ref-1)
2. Outpatient only [↑](#endnote-ref-2)
3. Inpatient only [↑](#endnote-ref-3)
4. Outpatient only [↑](#endnote-ref-4)
5. Inpatient only [↑](#endnote-ref-5)
6. Inpatient only [↑](#endnote-ref-6)
7. Inpatient only [↑](#endnote-ref-7)
8. Inpatient only [↑](#endnote-ref-8)
9. Outpatient only [↑](#endnote-ref-9)
10. Outpatient only [↑](#endnote-ref-10)
11. Inpatient only [↑](#endnote-ref-11)
12. Outpatient only [↑](#endnote-ref-12)
13. Outpatient only [↑](#endnote-ref-13)
