# Supplementary material for: Development of a novel observer-reported outcome measure for the assessment of Respiratory Syncytial Virus (RSV) infection symptoms in pediatric clinical trials
Source: J Patient Rep Outcomes. 2018 Feb 21;2:9. doi: 10.1186/s41687-018-0034-9 (PMC5935018; doi:10.1186/s41687-018-0034-9)
Supplement: Supplementary file 2 — Abbreviated Item Tracking Matrix. (DOCX 18 kb) [file 41687_2018_34_MOESM2_ESM.docx]

| Round 1 | Resulting Action | | Round 2 | Resulting Action | Final |
| --- | --- | --- | --- | --- | --- |
| Wheezing (OS: Overnight Symptom; DS: Daytime Symptom) | | | | | |
| OS/DS 1a. Wheezing severity    7 point categorical scale; 0-10 NRS | | **Based on participant feedback, combined with input from KOLs, this item was deleted following Round 1.** | N/A | N/A | N/A |
| Rapid or Shallow Breathing (OS: Overnight Symptom; DS: Daytime Symptom) | | | | | |
| OS/DS 2a. Rapid or shallow breathing severity with alternate wording options  7 point categorical scale; 0-10 NRS | | **Minor wording modifications were made and the alternate item dropped. The NRS scale option was removed.** | OS/DS 1. Rapid or shallow breathing severity  7 point categorical scale | **Minor wording modifications were made** | OS/DS 1. Rapid or shallow breathing severity  7 point categorical scale |
| Nighttime Cough Severity (OS: Overnight Symptom) | | | | | |
| OS 4a. Nighttime cough severity with alternate wording options  7 point categorical scale; 0-10 NRS | | **The alternate item was dropped. The NRS scale option was removed.** | OS 3. Nighttime cough severity  7 point categorical scale | **No changes were** **made to this item following Round 2.** | OS 3. Nighttime cough severity  7 point categorical scale |
| Daytime Cough Severity (DS: Daytime Symptom) | | | | | |
| DS 4a. Daytime cough severity with alternate wording options  7 point categorical scale; 0-10 NRS | | **The alternate item was dropped. The NRS scale option was removed.** | DS 3. Daytime cough severity  7 point categorical scale | **No changes were** **made to this item following Round 2.** | DS 3. Daytime cough severity  7 point categorical scale |
| Nighttime Cough Frequency (OS: Overnight Symptom) | | | | | |
| OS 5. Nighttime cough frequency  6 point VRS; 0-10 NRS | | **The NRS option was deleted following Round 1.** | OS 4. Nighttime cough frequency  6 point VRS | **No changes were** **made to this item following Round 2.** | OS 4. Nighttime cough frequency  6 point VRS |
| Daytime Cough Frequency (DS: Daytime Symptom) | | | | | |
| DS 5. Daytime cough frequency  5 point VRS; 0-10 NRS | | **The NRS option was deleted following Round 1.** | DS 4. Daytime cough frequency  5 point VRS | **No changes were** **made to this item following Round 2.** | DS 4. Daytime cough frequency  5 point VRS |
| Fever (OS: Overnight Symptom; DS: Daytime Symptom) | | | | | |
| OS/DS 6a. Fever severity with alternate wording options  7 point categorical scale; 0-10 NRS | | **The alternate item was dropped. The NRS scale option was removed.** | OS/DS 5. Overnight fever severity  7 point categorical scale | **No changes were** **made to this item following Round 2.** | OS/DS 5. Overnight fever severity  7 point categorical scale |
| Runny Nose (OS: Overnight Symptom; DS: Daytime Symptom) | | | | | |
| OS/DS 7a. Runny nose severity with alternate wording options  7 point categorical scale; 0-10 NRS | | **The alternate item was dropped. The NRS scale option was removed.** | OS/DS 6. Runny nose severity  7 point categorical scale | **No changes were** **made to this item following Round 2.** | OS/DS 6. Runny nose severity  7 point categorical scale |
| Stuffy or Congested Nose (OS: Overnight Symptom; DS: Daytime Symptom) | | | | | |
| OS/DS 8a. Stuffy or congested nose severity with alternate wording options  7 point categorical scale; 0-10 NRS | | **The alternate item was dropped. The NRS scale option was removed.** | OS/DS 7. Stuffy or congested nose severity  7 point categorical scale | **No changes were** **made to this item following Round 2.** | OS/DS 6. Stuffy or congested nose severity  7 point categorical scale |
| Nighttime Sleep (OS: Overnight Symptom) | | | | | |
| OS 9a. Nighttime sleep with alternate wording options  6 and 7 point VRS; 0-10 NRS | | **One of three alternate items were dropped. The NRS options were removed.** | OS 8a. Nighttime sleep with alternate wording options  7 point VRS | **Single item assessment of nighttime sleep was retained without changes following Round 2.** | OS 8. Nighttime sleep  7 point VRS |
| Activity Level (DS: Daytime Symptom) | | | | | |
| DS 9a. Activity level with alternate wording options  6 point VRS; 0-10 NRS | | **The alternate item was dropped. One of the VRS scale options was dropped. The NRS scale option was removed.** | DS 8. Activity level  6 point VRS | **In preparation for finalization of the items, revisions were made to change the response options so that the direction of the scale mirrored the other scales (i.e., no impact through severe impact). Additionally, the wording of the options was further modified to maintain consistency across the wording in the response scales included in the final version of the GRCD**. | DS 8. Activity level  6 point VRS |
| Disposition (OS: Overnight Symptom; DS: Daytime Symptom) | | | | | |
| OS 10a./DS 12a. Disposition (i.e., mood) with alternate wording options  6 and 7 point VRS options; 0-10 NRS | | **The alternate item was dropped. One of the VRS scale options was dropped. The NRS scale option was removed.** | OS 9/DS 11. Disposition (i.e., mood)  7 point VRS | **In preparation for finalization of the items, revisions were made to change the response option “Very much more fussy than usual” to “A great deal more fussy than usual” to maintain consistency across the wording in the response scales included in the final version of the GRCD.** | OS 9./DS 11. Disposition (i.e., mood)  7 point VRS |
| Daytime Sleep (DS: Daytime Symptom) | | | | | |
| DS 10a. Usual daytime sleep with alternate wording options  6 point VRS; 0-10 NRS | | **Because there was no clear majority, the option of “how much/long” was retained for further testing in Round 2. Additionally, the initial VRS was expanded to include response options that allowed for evaluation of decreased daytime sleep and the NRS option was deleted.** | DS 9. Usual daytime sleep with alternate wording options  11 point VRS | **While the expanded response category allowed participants to respond to the question, the two-tailed responses essentially measure two concepts and results could prove difficult to interpret. As the concept of increased sleep could be captured within the activity item (i.e., no activity) and decreased sleep could also correlate with mood (e.g., increased fussiness), this item** **was ultimately deleted from the final version of the GRCD.** | N/A |
| Eating Habits (DS: Daytime Symptom) | | | | | |
| DS 11a. Comparison to usual eating habits with alternate wording options  6 point VRS options; 0-10 NRS | | **One alternate item was dropped. The NRS scale option was removed from remaining two question alternates.** | DS 10a. Comparison to usual eating habits with alternate wording options  6 point VRS options | **Single item assessment of comparison to usual eating habits was retained without changes following Round 2.** | DS 10. Comparison to usual eating habits with alternate wording options  6 point VRS |

N/A = Not applicable due to item removal.

NRS = Numerical rating scale

VRS = Verbal rating scale
